# The effects of exercise training on knee repositioning sense in people with knee osteoarthritis: a systematic review and meta-analysis of clinical trials

**DOI:** 10.1186/s12891-023-06712-3

**Published:** 2023-07-19

**Authors:** Rahman Sheikhhoseini, Mahdis Dadfar, Shahnaz Shahrbanian, Hashem Piri, Mohammad Salsali

**Affiliations:** 1 Department of corrective exercise & Sport injury, Faculty of physical education and sport sciences, Allameh Tabataba’i University, Tehran, Iran; 2 Department of Human Health and Performance, Faculty of Kinesiology, University of Houston, Houston, TX USA; 3 Department of Sport Science, Faculty of Humanities, Tarbiat Modares University, Tehran, Iran; 4 Faculty of physical education and sport sciences, Allameh Tabataba’i University, Tehran, Iran

**Keywords:** Knee, Osteoarthritis, Proprioception, Exercises, Meta-Analysis

## Abstract

**Objective:**

Osteoarthritis (OA) of the knee is one of the most common global joint disorders, especially in aging population, and is among leading health-related concerns of societies. Therefore, this systematic review and meta-analysis was done to investigate the results related to the effects of exercise interventions on knee repositioning sense in patients with knee OA.

**Methods:**

An extensive search was independently performed in electronic databases including PubMed, MEDLINE, Web of Science, SCOPUS, and Google Scholar, to identify randomized clinical trials (RCTs) conducted on knee OA and to evaluate knee repositioning sense before and after different exercise interventions. After extracting relevant data from eligible studies, results of the studies were pooled using a random-effects model of meta-analysis. The Physiotherapy Evidence Database (PEDro) of clinical trials was used for quality assessment of eligible studies.

**Results:**

Among 2702 studies identified in the initial search, 17 studies were eligible for final systematic review and meta-analysis. The results showed that the patients who participated in different exercise interventions had significantly less knee repositioning error (mean differences: -1.141 degrees (95%CI: -1.510, -0.772, P < .001) compared to those who did not undergo exercise interventions. The eligible studies exhibited publication bias (Intercept: -6.69, P = .002), and the data showed significant heterogeneity (I2 = 85.633%, Q = 153.125, P < .001). Moreover, meta regression showed more prolonged exercise duration might have more effects on knee repositioning error (Coefficient=-0.860, 95% CI=-1.705, -0.016, Z=-2.00, P = .045).

**Conclusion:**

There is strong evidence that exercise interventions may effectively reduce knee repositioning error. Moreover, it seems that more prolonged exercise duration may be associated with the greater effect size.

**Supplementary Information:**

The online version contains supplementary material available at 10.1186/s12891-023-06712-3.

## Introduction

Osteoarthritis (OA) is one of the most common global joint disorders, especially in aging population, and is among leading health-related concerns in different communities [1]. In addition, financial burden of this disease has been estimated around tens of billions of dollars for governments to cure and tackle disease’s substantial consequences [2]. Knee OA is accompanied by various physical symptoms including pain, joint stiffness, limitations in physical activities, and loss of joint mobility [3]. It seems that, the OA patients’ walking quality and speed will be adversely influenced, and they will face with difficulties in ascending stairs and standing up from a chair [4]. In order to reduce the complications, several approaches are suggested for management of patients with knee OA [3, 5, 6]. Some of them include surgical approaches, such as arthroscopy and osteotomy and joint-replacement procedures [7]. There are also non-surgical treatments like orthotic devices [8] and improving individual’s lifestyle [3], of which exercise-based interventions are believed to play an essential role in management of patients with knee OA [9–11]. On the other hand, it seems that impaired proprioceptive sense of the knee may be present in patients with knee OA as well [12].

Proprioception plays a critical role in keeping humans҆ balance and movement control during daily activities. Any flaws in proprioceptive function may cause balance problems, leading to an increased risk of falling (9). It had been shown that different bodily processes, including muscular, sensory, cognitive, and psychosocial ones, change with aging. So, the loss of proprioceptive function is associated with aging in human beings [13]. Age-related loss of proprioception may alter the neuromuscular control of the limbs and the biomechanics of joints, impairing balance and increasing the risk of falls [3]. Moreover, it seems that the age-related loss of proprioception can be slowed by appropriate and proper physical activity [14]. In this line, some evidence suggests that knee repositioning sense may be impaired in patients with knee Osteoarthritis OA [12], and participating in exercise protocols may improve it (15). Previous studies have evaluated the effects of different exercise protocols on proprioceptive functions in patients with knee OA like Tai-chi and Baduanjin Qigong [16–18], balance and proprioceptive-based exercises [19–22], sensorimotor exercises [15, 23], computer-based exercises [24], Pilates [25], and different kinds of strengthening protocols [22, 26–28].

Previous studies have shown that exercise protocols may positively influence knee repositioning sense in patients with knee OA; however, there are controversial results regarding effectiveness of some exercise protocols [26, 29, 30]. So, it seems that running a systematic review with pooled meta-analysis would clarify strength regarding the effect of such exercises on repositioning sense in patients with knee OA. Therefore, this systematic review and meta-analysis was done to investigate the results on the effect of exercise interventions on knee repositioning sense in patients with knee OA.

## Materials & methods

### Search strategy of the literature

This study conducted in accordance with PRISMA guidelines [31]. The database of abstracts of reviews of effects (DARE) and the Cochrane Library were searched to find any systematic review and meta-analysis on this topic, but no systematic review and meta-analysis was found. Two independent investigators (MD and RS) performed an extensive review in electronic databases including PubMed, MEDLINE, Web of Science, and SCOPUS, and Google Scholar. A combination of the following keywords was used to search in the databases: [1] “sense of position”, “position sense”, repositioning, proprioception, [2] knee, hip, “lower extremity”, [3] osteoarthritis, osteoarthrosis, “degenerative joint disease”, and DJD. The “AND” and “OR” operators were used for between and within keyword groups, respectively. The search was performed from inception to 27 December 2022. All the searched citations with their abstracts were imported to the EndNote X7 software for more detailed checks.

### Selection criteria

Both reviewers (MD and RS) assessed the studies with respect to inclusion criteria. Only clinical trials that had administered any exercise training type for knee repositioning sense in the patients with knee OA were selected by screening titles and abstracts of the searched citations. Then, data on trial design and outcomes were extracted and summarized in a table. Any disagreements were resolved by consensus. The full texts of potentially eligible studies were retrieved and reviewed to determine whether they meet the inclusion criteria.

Only clinical trials that had been published in English peer-reviewed journals and had control groups were selected. There was no limitation about type of exercise training used or measurement tool to assess knee repositioning sense. Studies could be included regardless of repositioning sense being the primary or secondary outcome. Studies that had not included exercise groups or those that had combined exercise training with other interventions, such as manual therapy were excluded from the present research. The control group should not have performed exercise training, so studies, in which the control group had received no treatment or conventional treatments, were included. If any study had reported different measures for repositioning sense (e.g., different test angles, passive or active movements), the test results reporting the largest knee flexion range of motion -between 60 and 90 degrees- and active movements were only selected for data synthesis. No criterion was considered in terms of evaluation method and tests of the studies.

### Data extraction

The data extracted from eligible studies are summarized in Table 1. Table 1 shows the information including the first author names and years of publication, general characteristics of participants, available information about interventions and groups of study, quality assessment score of each study, the main utilized tools and methods, and the main results of the retrieved studies. The aim of the current study was to examine the effect of various exercise protocols on knee reposition sense.

**Table 1:** A Description of Eligible Studies

Study	Subjects		Exercise Training Protocol					
	Sample description	Sample Size (Men/Women) (Age ± SD)	Total duration of Exercise (Weeks, Frequency (d), Supervision)	Exercise Intervention Program	Control Intervention	Measured Variables	Main Outcomes	PEDro Scores
Sekir and Gur, 2005 [1]	Patients with bilateral knee grade 2 or 3 OA	E = 12 (3/9) (59 ± 8.9) C = 10 (3/7) (62 ± 8.1)	6, 2, NR	11 different balance/coordination and proprioception exercises including walking, stair exercises, and standings.	No exercise	Knee JPS (active and passive error at 20°, 45°, 70°), Kinaesthesia, Function (subjective rating activities), and Perceived knee pain (VAS).	Knee JPS, perceive knee pain and function were improved significantly	4/10
Tsauo et al., 2008 [2]	Patients with knee OA	E = 15 (1/14) (61.7 ± 6.6) C = 14 (4/10) (60.1 ± 6.7)	8, 3, NR	Exercising by a sling suspension system + physical therapy.	Routine physical therapy.	Active knee JPS (absolute error), Pain, Stiffness, and Function (WOMAC), functional tests (straight walking, figure-of-eight-walking, stair climbing).	Knee JPS and functional score were significantly improved	3/10
Jan et al., 2008 [3]	Patients with knee OA	E = 20 (5/ 15) (63.8 ± 8.3) C = 23 (7/16) (62.7 ± 7.8)	6, 3, Yes	Target-matching foot-stepping exercises	No exercise	Knee JPS, Functional incapacity score, and Walking velocity.	Knee JPS and functional incapacity score were significantly improved	5/10
Lin et al., 2009 [4]	Patients with knee osteoarthritis grade of 3 or lower	PrT = 36 (11/25) (63.7 ± 8.2) ST = 36 (12/24) (61.6 ± 7.2) C = 36 (10/26) (62.2 ± 6.7)	8, 3, NR	PrT: Participants sat and were asked to step on target pedals with a computer in various directions repeatedly. ST: Participants sat in a chair and performed full knee concentric extension based on quadriceps 1-RM	No exercise	Knee proprioception (absolute reposition error), Pain and Function (WOMAC), Walking time, Strength.	Knee proprioception was improved in both groups, while proprioception training group showed greater improvements. Pain and function were improved significantly in both groups.	8/10
Jan et al., 2009 [5]	Patients with knee OA	WB = 36 (12/24) (62.0 ± 6.7) NWB = 35 (10/25) (63.2 ± 6.8) C = 35 (11/24) (62.2 ± 6.7)	8, 3, NR	WB: participants performed knee extension fully and then flexion on an EN-dynamic resistance devise and in a sitting position. NWB: Participants were sited comfortably and performed knee flexion and maintained it with having the distal extremity in a free position.	No Exercise	Knee JPS (reposition absolute error), Function scale (WOMAC), Walking speed, Muscle torque.	Knee JPS was significantly improved in both groups, but more significantly in weight-bearing exercises group. Function was improved in both experimental groups.	7/10
Ahmed, 2011 [6]	Female Patients with knee osteoarthritis	E = 20 (all female) (60 ± 3.6) C = 20 (all female) (62 ± 3.2)	6, 3, NR	Sensorimotor training was performed at three static, dynamic, and functional stages from the easy to difficult movements + in addition to traditional exercise program.	Traditional exercise program: isometric and isotonic exercises	Knee proprioception accuracy, Pain (VNS), Function (arthritis impact functional assessment scale), Knee extensor muscle torque, Balance.	Knee proprioception, pain and function were improved significantly in both sensorimotor exercises and traditional exercises groups, but more significantly in sensorimotor group.	4/10
Duman et al., 2012 [7]	Patients with grade of 3 or higher knee osteoarthritis	E: 30 C: 24 (5/49) (64 ± 3.7)	3, 5, NR	Strengthening of quadriceps, ankle extensors and hip abductors, bicycling, zigzag walking in addition to anti-inflammatory drugs and physical therapy treatment.	Non-steroidal anti-inflammatory drug and physical therapy	Knee proprioception, Pain, Stiffness and Function (WOMAC), Balance (static and dynamic).	Knee proprioception was not improved significantly after proprioception exercises. However, WOMAC pain, stiffness and function were significantly improved after proprioceptive exercises.	2/10
Hale et al., 2012 [8]	Patients with mild to moderate OA and at risk for falling	E = 23 (6/17) (73.6 ± 1.5) C = 16 (4/12) (75.7 ± 1.1)	12, 2, Yes	Challenging balance exercises like different types of walking, jumping, leg swings, step-ups, in pair wrestling, etc., with both eyes open and closed with noodles, water dumbbells, weighted steps.	Time-matching computer training program	Knee proprioception and other physiological profile assessment	Knee proprioception was not significantly improved after aquatic exercises.	8/10
Schmid et al., 2013 [9]	Patients with knee OA. 75% female. With median K/L grade 4.	E: 16 (F: 80%) (63 ± 8.1) C: 17 (F: 70%) (68 ± 7.0 )	12, 2, Yes	Ten forms from classical Yan Style Tai Chi. Sessions programs were self-massage and reviewing principles of tai chi, tai chi movements, breathing technique, and relaxation.	received a wellness education and stretching Program	Knee proprioception (30◦, 45◦ and 60 ◦).	Knee proprioception was not significantly improved at 60◦, or at 45◦ in the intervention group. But it improved at 30◦.	7/10
Kumar et al., 2013 [10]	Patients with knee OA	Total: 44 (19/25) E = 22 (53.18 ± 6.88) C = 22 (53.32 ± 5.36)	4, 3, Yes	Conventional physiotherapy + knee extensors and flexors, hip flexors, extensors, abductors, and external rotators resistive training for + proprioceptive training	Conventional physiotherapy	Knee JPS (30◦, 45◦, 60◦), Pain (NRS), Function (WOMAC)	JPS was significantly improved in both proprioceptive exercise and conventional therapy groups, but more significant results were observed for proprioceptive exercising group. Pain and function were significantly improved in both groups.	6/10
Topp, and Pifer, 2017 [11]	Patients with knee osteoarthritis (one side more affected than the other)	D = 23 (6/17) (65.22 ± 2.34) I = 23 (9/14) (63.48 ± 2.19) C = 23 (5/18) (58.83 ± 2.81)	16, 3, 1session with supervision – 2sessions without supervision (at home)	D: 6 dynamic resistance bilateral exercises on ankle dorsiflexors and plantarflexors, knee and hip flexors and extensors. I: Subjects generated muscle tension without changing joints angles by therabands on ankle, knee, and hip flexors and extensors.	No exercise	Knee JPS (passively reproducing 30◦ flexion in both more and less affected knees), Time to detect passive movement (TTDPM)	Knee JPS in more affected knee was significantly improved in both groups, but isometric group showed higher improvements	5/10
Qingguang et al., 2017 [12]	Patients with Knee Osteoarthritis	E = 23 (all female) (64.6 ± 3.4) C = 23 (all female) (64.5 ± 3.4)	24, 3, NR	Eight classic Yang-style routine forms like stepping in multiple directions, weight shifting bilaterally, knee flexion extensions, dorsiflexion plantar flexions, and specific gait modifications.	Wellness education sessions.	Knee proprioception (flexion and extension in right and left knees), Pain, Stiffness and Function (WOMAC).	Knee proprioception, pain, stiffness and function significantly were improved	8/10
Vahid Mazloum et al., 2017 [13]	Patients with knee osteoarthritis	Total : 41 (25/13) P = 14 (55.0 ± 8.2) CT = 14 (50.3 ± 8.3) C = 13 (50.8 ± 9.9)	8,3, NR	P: exercises based on centering and focusing on spine and trunk, muscular control, performing exercises with precision and maintaining awareness, concentrating on exercises fully, breathing and flow and energy connection of all body parts during exercises. CT: exercises including buttock squeeze and rock, rock and stands half squats, stretching, standing balance, and home exercises routine.	Daily routine activity	Knee JPS (target angle reproduction), Pain (Lequesne index), Function (cumulative time for performing daily activities), and Disability.	Knee JPS was significantly improved in both Pilates and conventional therapeutic exercise groups, but no differences were observed between groups. Pain was significantly improved in both exercise groups, but Pilates group showed more significant improvements.	5/10
Raj et al., 2019 [14]	Patients with grade 2 and 3 knee OA	I = 15 (3/12) (53.67 ± 2.63) EB = 16 (4/12) (56.00 ± 3.52) CO = 15 (4/11) (56.80 ± 4.18) CT = 14 (3/11) (56.57 ± 3.67)	10, 2, NR	IG: Concentric and eccentric contraction for knee extensors and flexors EBG: Quadriceps isometric contraction, Hip adduction isometric, four ways straight leg raise and, 45 º knee extension COG: combination of above programs	Conventional therapy exercises	Knee JPS (active repositioning error at 45degree of knee flexion).	Knee JPS was significantly improved in all experimental groups. However, combination of isokinetic and EMG biofeedback showed to be more effective on proprioception.	4/10
Ye et al., 2020 [15]	Patients with knee osteoarthritis	E = 25 (12/13) (64.48 ± 7.81) C = 25 (8/17) (63.08 ± 3.65)	12, 3, NR	Baduanjin qigong exercises were based on Health Qigong-Baduanjin were done in 2 phases (at hospital and at home)	No exercise	Knee proprioception (ATE: average trace error in percentage, TTE: test time execution in seconds), Pain, Stiffness and Functional (WOMAC), Postural stability.	Knee proprioception, and pain and function were significantly improved	7/10
Chen et al., 2021	Patients with knee osteoarthritis	E = 16 (3/13) (60.31 ± 7.85) C = 16 (3/13) (60.94 ± 6.89)	4, 1, Yes (Straight leg raising, as a routine exercise, was prescribed to practice at home for both legs, 1 set of 10 repetitions, twice a day, and gradually increase exercise time to 3 sets over the 4-week period)	Taking part in Back Walking (BW), in addition the same treatment as the patients in the CG.	Straight leg raising, as a routine exercise.	Knee proprioception (ATE: average trace error, CT: completion time), Pain, Stiffness, Function (WOMAC and NRS)	Knee proprioception, pain, physical function and static stability were improved.	8/10
Shen et al., 2022	Patients with knee osteoarthritis	E = 14 (4/10) (65.3 ± 4.6) C = 13 (4/9) (66.6 ± 7.0)	6, 3, Yes	Proprioceptive neuromuscular facilitation (PNF) stretching	No exercise	Pain, Knee proprioception, Ankle proprioception, Range of Motion (ROM)	Knee and ankle proprioception, pain, balance and lateral compartment were improved.	7/10

Abbreviations: OA: Osteoarthritis, E: Experimental group, C: Control group, NR: Not reported, JPS: Joint Position Sense, VAS: Visual Analogue Scale, WOMAK: Western Ontario and McMaster Universities Osteoarthritis Index, WB: Weight Bearing, NWB: Non Weight Bearing, D; Dynamic, I; Isometric, VNS: Visual Numeric Scale, NRS: Numerical Rating Scale, TTDPM: Time to Detect Passive Movement, ATE: Average Trace Error, TTE: Test Time Execution, P: Pilates, CT: Conventional Training, IG: Isokinetic Group, EBG: Exercise with Biofeedback group, COG: Combination group,

### Quality assessment and level of evidence

The Physiotherapy Evidence Database (PEDro) of clinical trials and its guidelines were used for quality assessment of eligible studies. According to Sackett et al., level of evidence was also determined [32].

### Statistical analysis

The relevant data were extracted from eligible studies (including pretest and posttest means and standard deviations, P-values, sample sizes, and also possible mean differences and standard deviations). Then, results of the studies were pooled using a random-effects model of meta-analysis and the forest plot of the standard differences in means and estimated standardized Hedge’s g effect size with 95% confidence interval (CI). Q-test was applied to investigate data heterogeneity, while I^2^ was used to determine magnitude of possible data heterogeneity. In the case of data heterogeneity, meta-regression was run to identify the potential effect of exercise duration on meta-analysis results. Both funnel plot and Egger’s tests were used to check publication bias. The trim-and-fill method was used to evaluate the effect of adding possible random studies on the results if needed. Statistical analysis was performed using comprehensive meta-analysis (CMA) software version 3.0 (Biostat Inc, Englewood, New Jersey).

## Results

### Search results

A total of 2702 studies were found in the initial search. Nine hundred eighty-one duplicated citations were removed from the list. After reviewing abstracts/titles, 1681 studies did not meet the inclusion criteria, so they were excluded from the study. Then, the full texts of 40 studies were assessed in more detail. Again, 22 studies did not also meet the inclusion criteria.

**Fig. 1:**
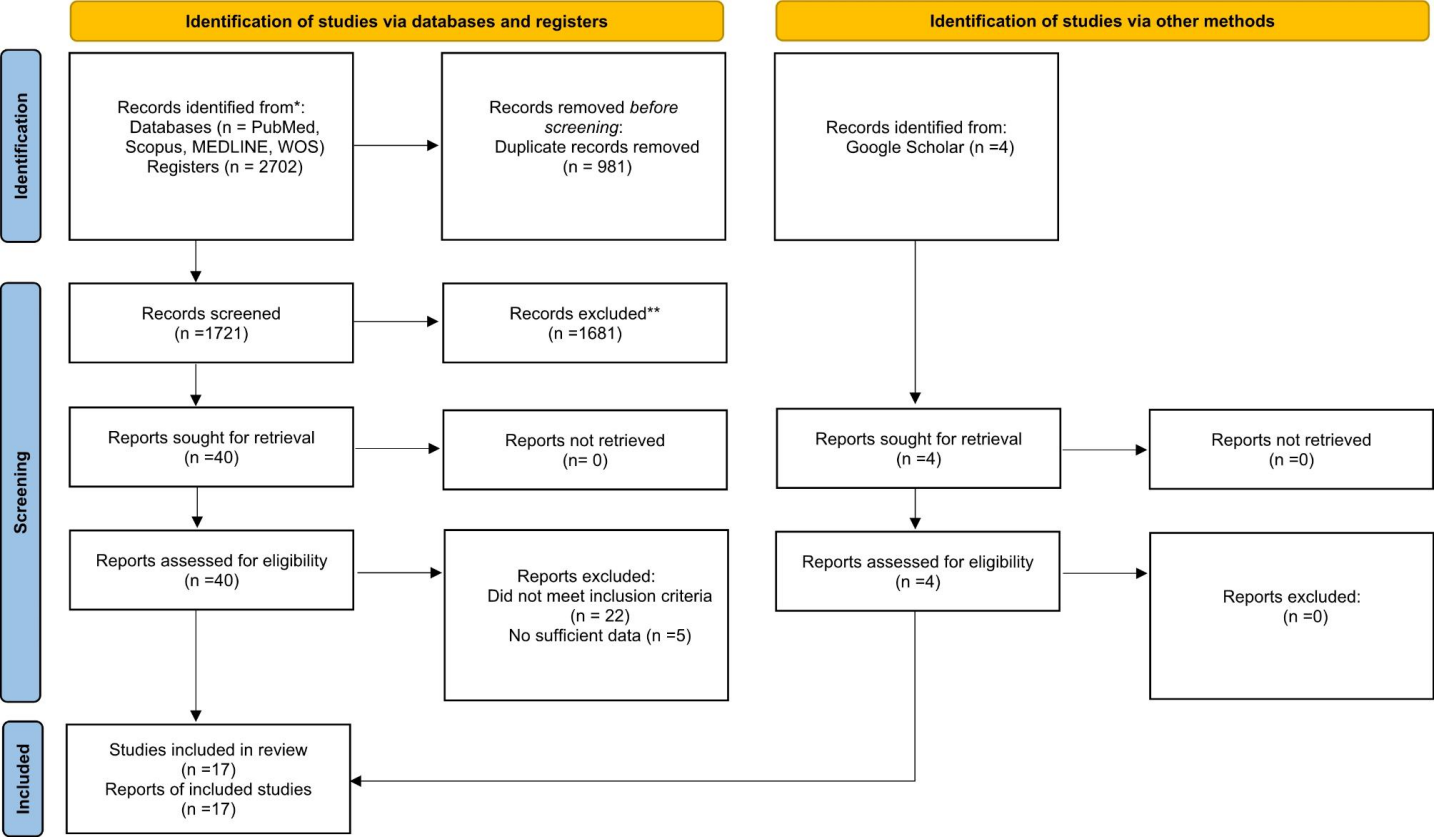
Flow diagram for eligible studies

Moreover, 5 studies had provided no sufficient data to be included in the study. Also, 4 studies were eligible for analysis from other sources including eligible studies obtained from citations and searches through Google Scholar database. Finally, 17 studies were selected to enter meta-analysis (Fig. 1). The information of eligible studies is summarized in Table 1.

### Study characteristics

Duration of the prescribed exercises ranged from 3 to 24 weeks, with 2–5 exercise sessions per week. Several types of exercise training had been prescribed in the studies including 3 studies on Thai-Chi and Baduanjin Qigong [16–18], 4 studies on proprioceptive exercises [19–22], 6 studies on resistance and strength training exercises [22, 26–28], 2 studies on sensorimotor training [15, 23], one study on Pilates [25], one study on aquatic therapy [33], one study on computer-based exercise [24], one study on backward walking [34], one study on proprioceptive neuromuscular facilitation [35], and 3 studies on other exercises including conventional therapeutic exercises, electromyographic (EMG) biofeedback, and a combination of EMG biofeedback and isokinetic exercises [25, 26]. Totally, 17 eligible studies had included 847 participants, as shown in Table 1.

### Description of the selected variables

Criteria for diagnosis of DJD of the Knee: Overall, 15 studies had used x-ray to evaluate knees [15–22, 24–28, 34, 35], and 2 studies had used clinical presentations only [23, 33]. Regarding knee OA severity, eligible studies recruited patients with different degrees of knee OA based on the Kellgren Lawrence scoring system. Patients with Grades 1–3 of knee OA had been examined in 5 studies [17, 22, 24, 27, 35], patients with Grades 2 and 3 of knee OA had been examined in 4 studies [15, 18, 19, 26], one study had investigated patients with Grades 3 and 4 of knee OA [20], one study had investigated patients with Grade 4 or lower of knee OA [25], one research had studied patients with Grade 4 of knee OA [21], one study studied the participants with at least grade 1 of OA [34], and 2 studies had not reported degree of knee OA [28, 33].

Knee repositioning error had been assessed using several tools including electronic goniometer in 3 studies [21, 22, 24], the CYBEX computerized dynamometer in one study [19], a custom-made JOBST air splint in one study [28], the Biodex system in one study [25], the Biodex 4 isokinetic device in one study [26], the shuttle mini-clinic device in one study [27], the CYBEX-NORM dynamometer in one study [20], the Prokin system in two studies [18, 34], the Biodex 3 pro-multi-joint isokinetic dynamometer in one study [23], Proprioception test devices [35], and the biometrics electro goniometer in one study [16].

### Data synthesis

In this meta-analysis study, meta-analysis was run using the standardized mean differences between intervention and control groups calculated in beginning and end of the exercise protocols for knee repositioning sense.

### Knee repositioning sense

**Fig. 2:**
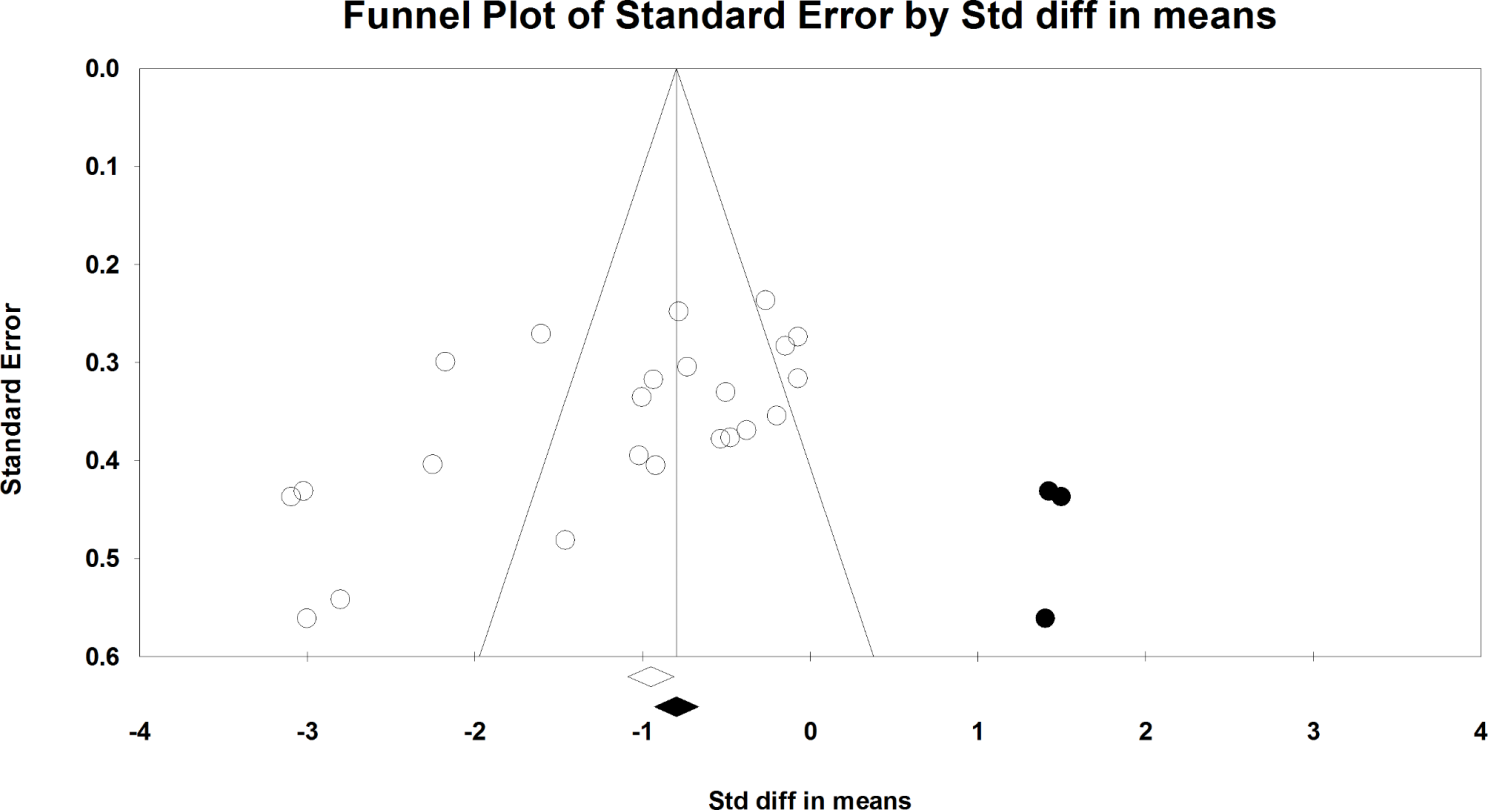
Forest plot regarding the effect of exercise on knee repositioning sense (favors **A**: Exercise, favors **B**: Control). CI: Confidence interval

**Fig. 3:**
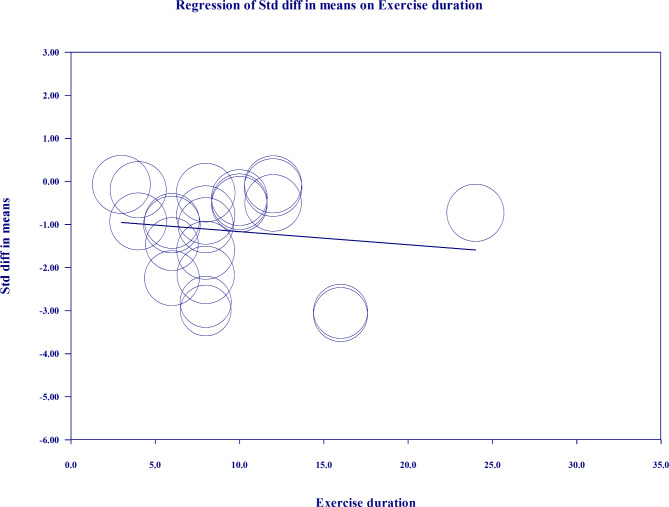
Funnel plot assessing the presence of publication bias in meta-analysis for knee repositioning sense. White-filled circles indicate the observed studies, while the gray-filled circles show the imputed studies

Meta-analysis was performed on 17 eligible studies [15–28, 33–35] that had obtained data on knee repositioning sense among a total of 847 participants with knee OA. The pooled estimated standard difference was − 1.141 degrees (95%CI: -1.510, -0.772, P < .001). The results showed that the patients who participated in different exercise protocols had significantly less knee repositioning error than control groups (Fig. 2). A funnel plot was used to assess the presence of the possible publication bias. Also, value of Egger’s test was significant (Intercept: -6.69, P = .002). The trim- and- fill method was used to investigate the effect of adding possible random publications to right side of the plot. The results showed that the main finding did not change, so it seems that publication bias might have no apparent effect on meta-analysis results (Fig. 3). Furthermore, significant heterogeneity was observed in data (I^2^ = 85.633%, Q = 153.125, P < .001).

**Fig. 4:**
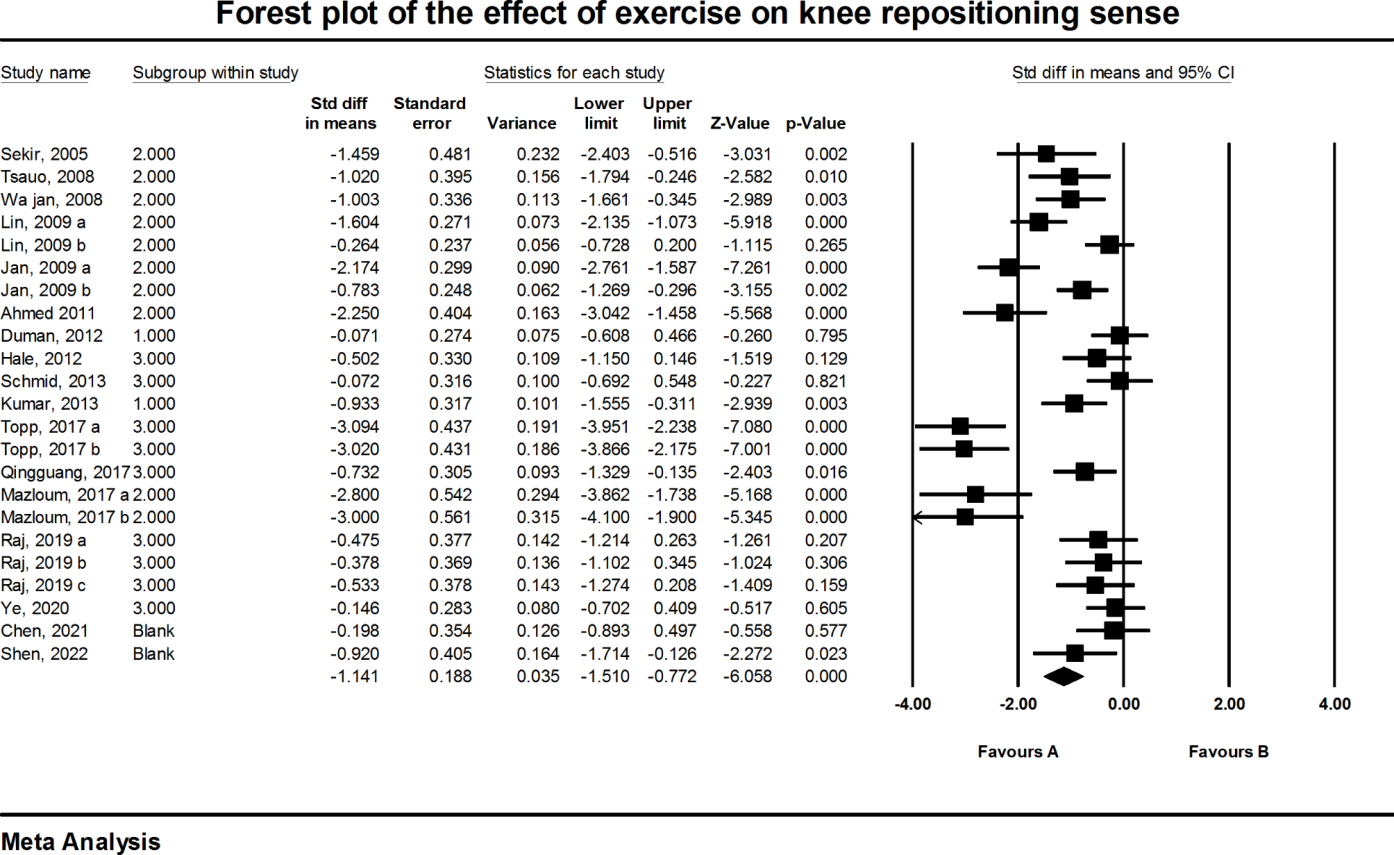
Meta-regression results that show longer exercise duration may have more effects on knee repositioning error

Possibly, the presence of data with high levels of heterogeneity may be attributed to different durations of the studies; so, meta-regression was run by adding the number of exercise training weeks as a covariate in meta-analysis. The results showed that the regression model was significant, and more prolonged exercise duration might have more effects on knee repositioning error (Coefficient=-0.860, 95% CI=-1.705, -0.016, Z=-2.00, P = .045). No significant associations were found between age, BMI, and OA severity variables and the effect size of exercise training in Meta regression to report (P > .05). The regression model is illustrated in Fig. 4.

As there were 6 high–quality studies [16–18, 21, 22, 27] showing the positive effects of exercise training on knee repositioning sense, strong evidence was assigned to this finding.

## Discussion

To the best of our knowledge, this study is the first systematic review and meta-analysis regarding the effect of different exercise interventions on knee repositioning sense in patients with knee OA. The results showed a strong evidence indicating that exercise intervention may be effective to decrease knee repositioning error (SD mean differences-1.141 degrees (95%CI: -1.510, -0.772, P < .001)).

According to our results, different exercise interventions can decrease knee repositioning sense in patients with knee OA. Moreover, it seems that more prolonged exercise duration may be associated with the greater effect size. It has been shown that mechanoreceptor contents of the knee meniscuses and ligaments are decreased in patients with knee OA [36]. On the other hand, knee repositioning sense not only relies on joint mechanoreceptors but also on periarticular tissues, such as tendons, capsules, and muscles [9]. So, it seems that an improvement in knee repositioning sense might result from muscle and neurophysiological adaptations to mechanical loadings [4] of different exercise regimens. Also, it appears that with more prolonged exercise duration, the body may have more chance to adapt to exercise [37], so showing the greater effect sizes in knee repositioning sense. Furthermore, our finding demonstrated that the effects of exercise therapy on proprioception was irrespective of age, BMI and OA severity. Generally, it seems that participating in any exercise program may improve knee repositioning sense in patients with knee OA of various ages, BMIs, or OA severity.

With regard to the existing heterogeneity among the eligible studies, as mentioned in the methods section, different tools were used for examining knee reposition sense. But the scale of all of them was in degrees. So standard differences in means were used to pool data. But the accuracy and reliability of various tools are different in examining the knee repositioning sense [38–40]. On the other hand, various exercise protocols were prescribed in eligible studies. We should remember that various exercises may have different impacts on knee repositioning sense in knee OA patients. Moreover, the participants had OA of varying severity. It is also to be expected that the different severity of the disease may impact the change in joint reposition sense. Accordingly, it seems that the different measurement tools and the prescribed exercises may result in heterogeneity among eligible studies.

The findings of this study should be evaluated with respect to several limitations. Firstly, the study included only original papers in English peer-reviewed journals. Here, a strong level of evidence was found regarding the positive effects of exercise therapy on knee repositioning sense. Still, high levels of data heterogeneity highlight the need for caution in generalizing and interpreting these results to all forms of exercise or all exercise durations. This study protocol has not been prospectively registered in advance in any registration database. Finally, the eligible studies were included regardless of repositioning sense being the primary or secondary outcome, so it should be remembered that included studies are not being powered to show improvement in repositioning sense.

## Conclusion

The results showed strong evidence stating that exercise intervention might reduce knee repositioning error. Moreover, it seems that more prolonged exercise duration may be associated with the greater effect size. Because of high levels of data heterogeneity among the reviewed studies, more studies are needed to clarify the effects of different types of exercise or durations on patients with knee OA.

## Electronic supplementary material

Below is the link to the electronic supplementary material.

Supplementary Material 1

## Data Availability

The datasets generated and analysed during the current study are available in the supplementary file 1 in the journal.
